# Climate Change Expands the Spatial Extent and Duration of Preferred Thermal Habitat for Lake Superior Fishes

**DOI:** 10.1371/journal.pone.0062279

**Published:** 2013-04-26

**Authors:** Timothy J. Cline, Val Bennington, James F. Kitchell

**Affiliations:** 1 Center for Limnology, University of Wisconsin, Madison, Wisconsin, United States of America; 2 Center for Climatic Research, University of Wisconsin, Madison, Wisconsin, United States of America; The Australian National University, Australia

## Abstract

Climate change is expected to alter species distributions and habitat suitability across the globe. Understanding these shifting distributions is critical for adaptive resource management. The role of temperature in fish habitat and energetics is well established and can be used to evaluate climate change effects on habitat distributions and food web interactions. Lake Superior water temperatures are rising rapidly in response to climate change and this is likely influencing species distributions and interactions. We use a three-dimensional hydrodynamic model that captures temperature changes in Lake Superior over the last 3 decades to investigate shifts in habitat size and duration of preferred temperatures for four different fishes. We evaluated habitat changes in two native lake trout (*Salvelinus namaycush*) ecotypes, siscowet and lean lake trout, Chinook salmon (*Oncorhynchus tshawytscha*), and walleye (*Sander vitreus*). Between 1979 and 2006, days with available preferred thermal habitat increased at a mean rate of 6, 7, and 5 days per decade for lean lake trout, Chinook salmon, and walleye, respectively. Siscowet lake trout lost 3 days per decade. Consequently, preferred habitat spatial extents increased at a rate of 579, 495 and 419 km^2^ per year for the lean lake trout, Chinook salmon, and walleye while siscowet lost 161 km^2^ per year during the modeled period. Habitat increases could lead to increased growth and production for three of the four fishes. Consequently, greater habitat overlap may intensify interguild competition and food web interactions. Loss of cold-water habitat for siscowet, having the coldest thermal preference, could forecast potential changes from continued warming. Additionally, continued warming may render more suitable conditions for some invasive species.

## Introduction

Species distributions are predicted to shift for many plants and animals as a result of climate change [1]–[4]. Global air temperatures are expected to increase 1.4 to 5.8°C over the next century [5]. Temperature is an ecological master factor and defines habitat bounds, niche space and species interactions [6]–[9]. As the climate warms, availability of preferred thermal habitats can change in both extent and duration. Ectotherms, like fish, are particularly sensitive to temperature regimes [2]. In general, fishes are grouped in warm, cool, or cold-water guilds based on their realized thermal niche [7]. Climate change is likely to alter fish distributions, growth potential, fecundity, and trophic interactions [8], [10], [11]. However, variation in the thermal regime is not likely to affect each of the guilds symmetrically.

Aquatic systems at higher latitudes may be particularly vulnerable to warming as their temperatures can rise faster than air temperatures due to reduced ice cover, decreased albedo effects, and earlier stratification [12]–[14]. Lake Superior has recently been recognized as one of the most rapidly warming lakes in the world [13], [15]. Summer (July-August) lake surface temperatures have increased 2.5°C between 1979 and 2006, increasing the length of the stratified period [15]. Lake Superior has large volumes of cold water and contains abundant populations of species belonging to the cold and cool water guilds. While warm water fishes are largely restricted to shallow bays, climate change may open main lake areas and shift cool water fishes toward deeper mid-lake habitats. Rising temperatures could contribute to reorganization of the dominant members of the fish community.

Intense commercial fishing, habitat losses owing to deforestation, and invasion by the sea lamprey (*Petromyzon marinus*) caused major changes in ecosystem structure and function for each of the Laurentian Great Lakes 16,17. Native lake trout and many other fishes were extirpated in the lower lakes (Ontario, Erie, and Michigan). In Lake Superior, extensive declines of lake trout occurred during the 1950s [18] but remnant populations persisted and joint US/Canada management actions developed in pursuit of restoration goals [19]. Through fisheries restriction and successful control of sea lamprey, resource managers have largely restored Lake Superior native fish populations. Commercial fisheries for lake trout have been partially restored and billion-dollar recreational fisheries for lake trout and introduced Pacific salmon now flourish [19], [20].

Lake Superior is currently the only Laurentian Great Lake with intact native fish populations 20,21. Total ecosystem function is at or near historic structure and productivity levels [22]. Restoration and preservation of the native fish community is a primary focus for Great Lakes fisheries management [20], [21]. While management focuses upon stabilization of native fish species within the lake, key habitat elements are changing each year and over time as climate change develops.

How significant were the changes in temperature experienced by the fish in Lake Superior? Austin & Colman [13] used three mid-lake buoys operated by the National Buoy Data Center to show that Lake Superior surface water temperatures were increasing rapidly starting around 1980. While this work was pivotal, isolated mid-lake temperatures do not provide information about the spatial extent of preferred habitat available to each of the fish guilds, how this extent varies, or the duration of preferred thermal habitat experienced by fishes in different parts of the lake. Mid-lake buoys provide critical observations of lake temperatures and atmospheric conditions at three lake points from spring to fall. But, most fish species reside near shore or in deep water demersal habitats where observations are sparse and/or not continuous.

There have been many theoretical or predictive studies forecasting the effects of climate change on fish thermal habitat for 20 to 100 years into the future based on varying climate model predictions [11], [23], [24]. There are few direct reports of changes in preferred thermal habitat of fishes based on known data from climate warming effects on a whole lake ecosystem. Lake Superior is of particular interest because it’s the world’s largest freshwater lake ecosystem in spatial extent. Here we use a three-dimensional hydrodynamic model of Lake Superior to investigate the spatial structure of preferred thermal habitats for two lake trout ecotypes, lean and siscowet, Chinook salmon, and walleye. We present these results for the period of 1979–2006 where atmospheric climate data are fully available. These data are essential for applying the hydrodynamic model to forecast water temperatures [25]. In 1979, lake warming was generally beginning and by 2006 temperatures were substantially warmer and typical of the past five years [13], [15]. We use the model to provide lake-wide maps of the number of days with preferred thermal habitat for each species. We quantify changes in preferred habitat extent, both as inter-annual variability and long-term trends. We discuss the potential implications for shifting species distributions, changes in ecological interactions and fisheries management into the future.

## Methods

### Study System

Lake Superior is a very large, deep, ultra-oligotrophic lake in North America. It has a surface area of 82,100 km^2^. It is the largest lake on Earth by surface area and third largest by volume. Lake Superior has a mean depth of about 150 m and a maximum depth of 407m. During winter 10 to 40% of Lake Superior’s surface is covered by ice [26]. Mean summer surface temperatures range from about 6°C to about 12°C [15]. Over the last three decades ice cover has declined and surface temperatures have risen [13], [15]. Lake Superior has 86 known species of fish. It contains healthy populations of most of its 71 native fish species, but also has populations of 15 non-native fish species [21]. Conservation of Lake Superior’s native fish community is a management priority [19].

### Hydrodynamic Model

The Massachusetts Institute of Technology general circulation model (MITgcm) [27], [28] was configured at 2 km horizontal resolution for the bathymetry of Lake Superior [29] and extensively evaluated by Bennington *et al.* [25]. The hydrodynamic model has 28 vertical layers. The upmost ten layers are each 5 m thick and then increase in thickness with depth to a maximum thickness of 33.7 m at 322 m depth. Sub-grid scale processes were estimated using vertical mixing [30] and horizontal diffusion [31] schemes [25].

An atmospheric module uses bulk formulae to calculate exchange of heat, moisture, and momentum between the atmosphere and the lake, dependent on atmospheric stability. Observations of lake ice coverage [26], [32] are applied as fractional coverage to each model grid cell at daily resolution, thus eliminating the need for a lake ice model. Lake albedo, evaporation, heat and momentum exchange are all reduced by the fraction of ice cover present.

The model utilizes above-lake atmospheric conditions determined by the North American Regional Reanalysis Project (NARR) [32]. Three hourly winds, downward shortwave and longwave radiation at the surface, air temperature, and specific humidity from 1979–2006 force the model. Ice observations were only available through 2006 at model run time. The model is spun up for five years using 1979 forcing before the inter-annual run is completed. The model outputs daily average temperature and currents for all model grid cells (304×148×28 cells, longitudinal, latitudinal, and vertical, respectively) for the entirety of the model run. Biases within the NARR atmospheric product due to lake surface boundary conditions cause a warm bias in the lake hydrodynamic model, enhanced during spring and cooler years [25]. This causes the model to stratify earlier than observed. Mean temperature biases (2–4°C) between April and November are due to this early stratification, when model error is largest. The model overestimates summer temperatures during cold years, but not during warmest years [25]. Thus, modeled habitat extents are overestimated more significantly during cooler years. Compared to summer temperature trends at the buoys, the model only captures half of the observed increase, thus historical modeled trends in habitat are conservative estimates. The model overestimates temperatures during the early part of the period and underestimates inter-annual variability. Model fit to observed temperature data is fully explored and described in Bennington et al. 2010 [25].

To assess observed water temperature for the years beyond the model we extracted mid-lake buoy hourly surface water temperatures for 1981–2011 from the National Oceanic and Atmospheric Administration National Buoy Data Center (http://www.nbdc.noaa.gov/). Mean summer temperatures for each year were computed as the arithmetic mean of all measurements from 1 June to 30 September of each year.

### Fish Thermal Preferences

The fundamental thermal niche, herein called ‘preferred’ thermal habitat, of a fish species has been defined as ±2°C from the median preferred temperature [7]. Maximum growth occurs at temperature within this thermal niche if food is readily abundant [7]. Above the greater extreme, respiration rates are greatly elevated and can outpace consumption rates. In some cases, this can approach lethal thermal tolerances, which strongly limits habitat extent [6], [7]. Below the lesser extreme, consumption and respiration rates slow, resulting in reduced growth [6]. Optimal temperatures for consumption are often measured in laboratories and applied to bioenergetics models [6]. These optimal temperatures may not precisely correspond with preferred temperatures in natural settings as realized temperatures represent tradeoffs between physiological temperatures, available food, and competition avoidance. However, thermal preferences in field studies are often closely related to energetically optimal temperatures [33], [34]. When available we used *in situ* preferred temperatures derived from field studies, which balances these competing interests. Lean lake trout have a preferred temperature of 10°C measured by thermal tagging in Lake Superior [34]. Siscowet lake trout have a preferred temperature of 4°C based on hydroacoustic measurements of habitat occupancy in Lake Superior [35]. There was no thermal tag information for Chinook salmon in Lake Superior. Energetic models developed for Chinook salmon use 11°C [36], but in Lake Ontario they have been observed above 14°C [37]. We employed a preferred temperature of 13°C for Chinook salmon, a compromise between these two [38]. For walleye we used a preferred temperature of 21°C [39].

When surface waters are at or below 4°C, the lake is either un-stratified or reversely stratified. Once the lake stratifies, lake temperatures are the warmest in the upper mixed layer and generally decrease over depth with a thermocline near 20 m where temperature declines rapidly to about 4°C. In shallow water regions, the entire water column may be warmer than preferred temperatures for some fish species. Thus, we determine whether the preferred temperature habitat is available within the water column of each model grid cell each day by examining the entire water column. If preferred temperature (Tpref) ±2°C is available anywhere within the column, the location is deemed suitable as preferred habitat for that day. Habitat volume is approximated as horizontal spatial extent of preferred temperatures. Observations are insufficient to determine whether mixed layer depths have changed in the lake. Increases in surface temperature may strengthen stratification, while increases in wind speeds work to decrease the mixing depth. Bennington *et al.* [25] found no statistically significant change in mixed layer depths over the period of interest. We assume that changes in mixed layer depths do not contribute to habitat change therefore spatial extent should be strongly correlated with volume.

We determine the magnitude of inter-annual variability by calculating the standard deviation of days with preferred temperature for each model grid cell. We calculate a linear trend in days with preferred temperature across the lake using an ordinary least squares regression. Given the model underestimates actual trends, we reject the null hypothesis that there is no change in the number of days with preferred temperature for a lake point and species if the probability of a trend is greater than 90% (α = 0.10). To determine habitat expansion through time, we examine the spatial extent of the lake with at least the historical (the first 3 years of the model) median number of days with preferred temperature for each species. This corresponds to finding the total lake area in each year with at least 330, 140, 106, and 11 days with available preferred temperature, for siscowet, lean lake trout, Chinook salmon, and walleye, respectively. These durations give a benchmark to compare the total area within the lake that experiences an ‘average’ amount of thermal habitat with each year.

## Results

**Figure 1:**
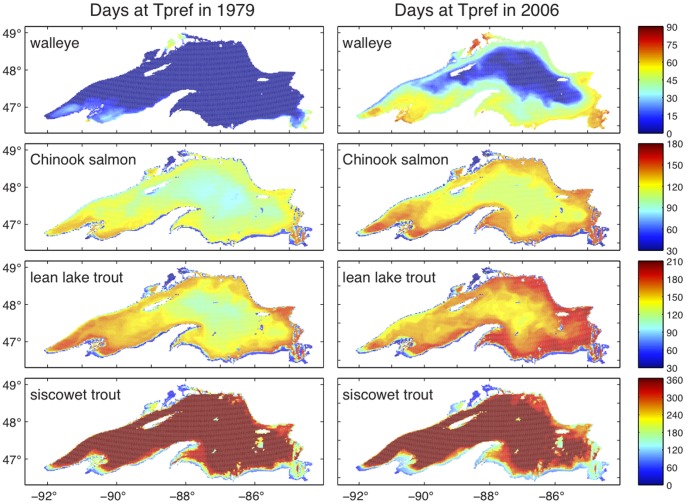
Spatial extent and duration of preferred thermal habitat in contrasting years. Days with available preferred temperature are shown for walleye, Chinook salmon, lean lake trout, and siscowet trout in 1979 and 2006 at all modeled lake points. Preferred temperatures for walleye, Chinook salmon, lean lake trout, and siscowet trout are 21(±2°C), 13(±2°C), 10 (±2°C), and 4 (±2°C), respectively.

Preferred thermal habitat extent of the four fishes is not stationary in time. Figure 1 displays preferred thermal habitat of walleye, Chinook salmon, lean, and siscowet lake trout across the lake as the number of days each fish species can find a depth with temperature equal to Tpref ±2°C for two contrasting years. The left column shows the preferred habitat for 1979, the first year simulated and near the long-term temperature average [15]. The right column illustrates available days for Tpref ±2°C in 2006, the last year of the simulated period. During the cold year of 1979, the preferred thermal habitat for walleye (top row) is restricted to the shallow waters near the southern shore in the western arm of the lake and within small, shallow bays of the northern coast. For 2006, habitat duration for walleye has increased in these near shore areas and preferred habitat significantly expands around the entire lake, except in the deepest portions and in the coastal upwelling zone on the northern side of the western arm. On average, walleye had an additional month of preferred thermal habitat available during 2006, as compared to 1979.

Chinook salmon (second row, Figure 1) preferred thermal habitat extends lake-wide, even in a cold year. The number of days with preferred thermal habitat is considerably less in the deeper lake regions. Eastern bays provide the greatest number of days with preferred thermal habitat during the cold 1979. In general, Chinook salmon thermal habitat is greatest just offshore, where the waters are deep enough so as not to overheat during summer, yet shallow or close enough to shore to be warmed by the counterclockwise circulation that brings warmer waters from the southern part of the lake to the north. During 2006, the duration of days with preferred thermal habitat lengthens by approximately a month throughout the lake for the Chinook salmon, except in the shallow, very near shore zones where waters become warmer than preferred.

Lean lake trout (Figure 1, third row) could find preferred thermal habitat for at least 3 months anywhere in the lake during 1979. As with Chinook salmon, waters immediately offshore can become warmer than preferred during the summer and optimal habitats exist just offshore. The coastal upwelling (northwest coast) and very shallow waters immediately off the southern shore do not offer preferred thermal habitat in 2006. Despite extensive preferred thermal habitat available in 1979, the days with preferred thermal habitat increase lake wide by approximately one month for lean lake trout in 2006. Siscowet lake trout have the largest and longest duration of thermal habitat with only shallow near shore areas reaching the high end of their thermal niche (Figure 1, bottom row). However, siscowet lake trout lose days of preferred thermal habitat near the coast in the warmer 2006 (Figure 1, bottom row). Preferred thermal habitat varies significantly from year-to-year. Each fish species may inhabit new lake regions in warmer years (Figure 1).

**Figure 2:**
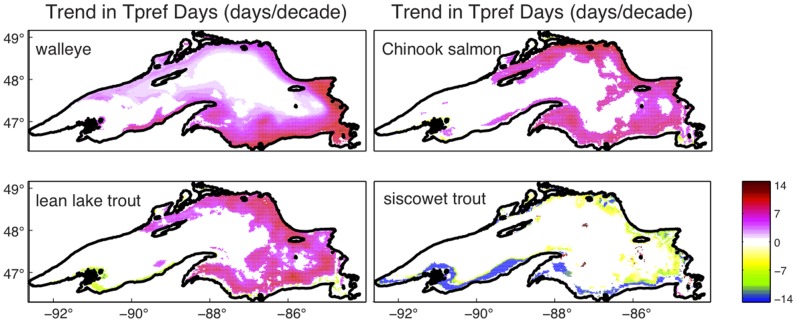
Spatially explicit trends in the duration of preferred thermal habitat. Trends in the number of days (days•decade^−1^) when preferred temperatures are present between 1979–2006 for all modeled lake points. Trends were computed using ordinary least squares regression using α = 0.10 (see methods). White indicates no significant trend in growing days.

We represent spatial extent of preferred thermal habitat changes over time as the linear trend in days with preferred thermal habitat for each lake point over the period of 1979–2006 (Figure 2). Only statistically significant trends are shown in color. For walleye there was a general increase in days of preferred temperature in near shore waters along the southern shore and in the eastern basin. The largest increases of nearly two weeks per decade developed in the southeastern basin. In the northern bays temperature increases provided an additional week per decade of preferred thermal habitat between 1979 and 2006. No significant trends were found within the deepest portions offshore or in the southwestern arm of the lake.

Both lean lake trout and Chinook salmon also experienced a statistically significant increase in days of preferred thermal habitat within about 25 km of shore in the eastern and northern portions of the lake. In these warming regions, trends for the Chinook salmon and lean lake trout were greater than 7 days per decade between 1979 and 2006. Near the southwestern shore both the Chinook salmon and lean lake trout habitats exhibited a statistically significant decrease in the number of days with available preferred thermal habitat due to warming of the water column above the preferred temperature range for each species. Lean lake trout experienced a greater loss of days with preferred habitat near the southwestern shore than did Chinook salmon. Siscowet trout are the only fish species here to lose days of preferred thermal habitat around the entire lake, except in the upwelling region of the western coast, where duration of preferred thermal habitat remains unchanged.

**Figure 3:**
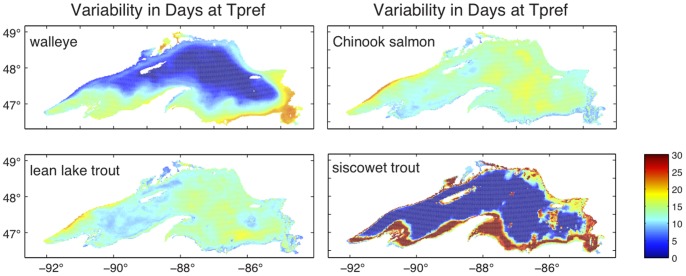
Interannual variability in preferred thermal habitat. Variability is shown as the standard deviation in days with available preferred habitat across all years from 1979 to 2006.

Overall the trends in days with available preferred thermal habitat are smaller than the year-to-year variability in these values. Standard deviations in days of preferred thermal habitat for the four fishes are presented in Figure 3. Of the four fishes, walleye experience the highest level of interannual habitat variability (integrated lake wide) and greatest overall increase in habitat expansion. Most of that occurs in the southern and southeastern shores and in bays of the northern shore. Siscowet trout experience significant coastal variability and almost no open lake variability, due to the open lake depth. Lean lake trout and Chinook salmon experience a more homogenous interannual variability in spatial extent and duration of preferred thermal habitat. Along the northwestern shore, changes in coastal upwelling from year to year results in highly variable duration of preferred thermal habitat.

**Figure 4:**
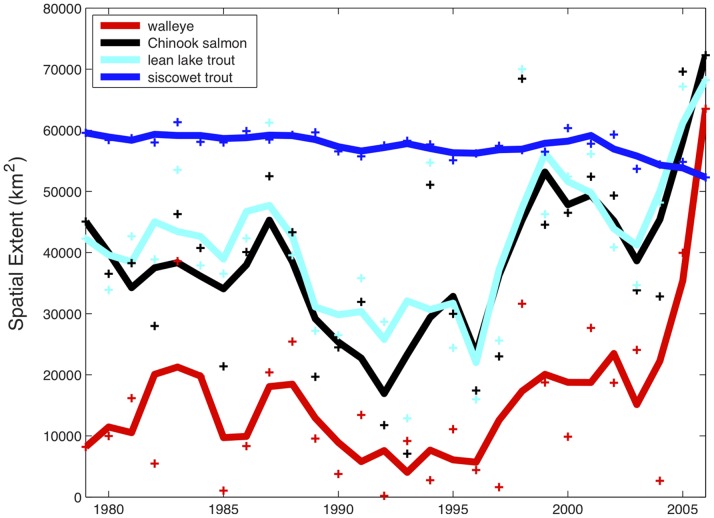
Areal extent of preferred thermal habitat over time. Areal extent is computed as the total surface area in each year that contains at least the historical (first three years) median number of days with preferred thermal habitat available for walleye, Chinook salmon, lean lake trout, and siscowet trout between 1979 and 2006. The threshold number of days for walleye, Chinook salmon, lean lake trout, and siscowet trout are 11, 106, 140, and 330 days respectively (see Methods). Solid lines depict three year running mean.

Lake wide, total spatial extent of preferred thermal habitat increased for walleye, lean lake trout, and Chinook salmon. Total spatial extent of median number of days with available preferred thermal habitat is represented in Figure 4. For walleye, preferred thermal habitat extent has increased three fold since the early 1980s and sharply increased in the 2000s. Spatial habitat extent for Chinook salmon and lean lake trout changed modestly in the 1980s before increasing during the period of mid-1990s to 2006. Both Chinook salmon and lean lake trout preferred thermal habitat exhibit greater than 50% increase from that of the early 1980s. Spatial extent of preferred thermal habitat for siscowet lake trout was relatively stable from 1979–1999 but declined 13% from 2000–2006.

## Discussion

A hydrodynamic model with high spatial resolution revealed substantial global climate change effects on available preferred thermal habitat for four fishes in Lake Superior. We find that duration and spatial extent of preferred thermal habitat for walleye, Chinook salmon, and lean lake trout, has increased significantly between 1979 and 2006, while siscowet trout have been forced to move further from the coast. This follows a steady rate of warming in the surface waters of Lake Superior beginning around 1980 [15]. Similar to the effects of climate change worldwide, we find a significant trend in inter-annual variability.

Lake Superior has abundant populations of both lean and siscowet lake trout, and of naturalized salmon [20]. Siscowet lake trout have reached abundances five times greater than lean lake trout [40]. This could be, in part, due to 50% greater spatial extent and longer durations of cold-water habitat. As warming alters preferred habitat in space and time, the relative amounts of preferred habitats for these species is shifting and spatial extent of preferred habitat for lean lake trout now exceeds that of siscowets.

Magnunson *et al.* [23] used climate-forecasting scenarios to project changes in preferred thermal habitat in Lake Michigan. They found that cold and cool water fishes would lose habitat under climate warming scenarios. Lake Michigan and Lake Erie are significantly warmer than Lake Superior and are likely experiencing large areas where the water columns are warming above the thermal preferences of the cool water fish guilds. In Lake Superior, for lean lake trout, Chinook salmon, and walleye, warming has expanded preferred thermal habitat. In contrast, cold-water siscowet lake trout habitat extent was reduced as the water column in the near shore area warmed to exceed the preferred thermal temperatures. This is evident in the decrease in available habitat in the near shore areas for siscowet (Figures 1, 4). Future warming will likely continue to exclude cold-water fishes from near shore habitats and may eventually begin to shrink cool-water fish habitat as well. The results from analysis of the lower Great Lakes may provide insight for the future of thermal habitat in Lake Superior.

Available preferred thermal habitat is one of several factors that could determine productivity for predatory fishes. Christie & Regier [41] found strong positive trends between the spatial extent of optimal thermal habitat and sustainable fishery yields for four fish species including lake trout and walleye. Competition and prey availability will influence whether predators will be able to achieve the increased levels of consumption allowed by increased preferred thermal habitat. Kitchell *et al.* [17] showed that production of forage fish is a key constraint for all salmonids (trout and salmon) in Lake Superior. In the western arm, Chinook salmon growth rates have declined dramatically since the 1990’s as a result of increased predator density and reduced per capita prey availability [22]. The major pelagic prey fish species, rainbow smelt (*Osmerus mordax* and *Coregonus* spp.), have similar preferences to the cold-water predators lake trout and cool-water Chinook salmon. Therefore habitat expansion is occurring for prey fish as well and could potentially improve their populations. On the other hand, there is no evidence of increased productivity at lower trophic levels in this ultra-oligotrophic lake and the general increase in temperature-dependent predation rates may have negative effects on prey populations.

For most stratified inland lakes and estuarine areas, thermal stratification during summer prevents mixing of dissolved oxygen from the upper oxygen rich layers to the bottom of the water body where metabolism exceeds production. This creates an anoxic or hypoxic environment inhospitable for fish or zooplankton. While the duration and spatial extent of available preferred thermal habitat is increasing, thermo regulation by fish requires them to seek deeper waters as the surface warms. A consequence of these opposing physiologic limitations is a constriction between warming surface waters and the low oxygen hypolimnion. In Lake Superior, long windblown distances allow for deep physical mixing, preventing hypoxia [42]. Therefore the constriction in physical and chemical habitat requirements may not limit available habitat for these predatory fish in this system.

**Figure 5:**
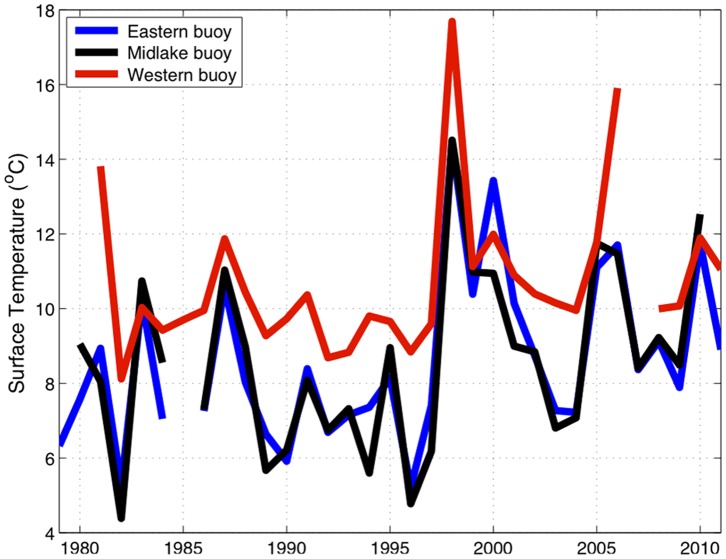
Observed mid-lake summer water temperatures in Lake Superior. Temperatures reported are the average observed mean summer surface water temperatures (1 June through 30 September) at the eastern, middle, and western Lake Superior NOAA buoys from 1981–2011.

Although the model results here are limited to 2006 and prior years, buoy observations from 2007–2011 show the warm temperatures in 2005 and 2006 that help drive trends in this work are not anomalous (Figure 5). Years 2007–2011 are some of the warmest summers recorded by the buoys. As Lake Superior continues to get warmer, managing for cool and cold water native fishes may become more challenging. Additionally, Lake Superior is warming asymmetrically [25], [38]. The majority of change in preferred thermal habitat is occurring in the eastern half of the lake (Figure 2). Much of the monitoring effort is directed in the western arm. More effort should be placed on monitoring the eastern waters where rapid change is most apparent.

Aquatic invasive species are a major concern worldwide. The Laurentian Great Lakes have more than 180 known invaders [43]. Climate change is expected to alter the effectiveness of natural filters for preventing species invasion [44]. Climate warming in Lake Superior may improve the habitat suitability for invaders with warmer thermal preference.

Sea lamprey (*Petromyzon marinus*), a voracious invader in the Great Lakes, has long been established in Lake Superior, has a median thermal preference around 18°C [45], and is currently managed at 5–10% of former abundances while abundance of host species has increased several-fold 20,22. The realized thermal temperature for sea lamprey depends on the thermal preferences of its host species. As a result, the extended duration and extent of preferred thermal habitats for the dominant host species, lake trout and salmon, will increase parasitic feeding, growth, and fecundity of lampreys presenting management challenges for the future [38].

Lake Superior’s fish community is monitored through various federal and state government and first nation organizations such as US fish and wildlife, US Geological Survey, Ontario Ministry of Natural Resources, and the Great Lake Indian Fish and Wildlife Commission. From the most recent State of Lake Superior report lean lake trout catch-per-unit-effort generally declined between 2001 and 2005 in the western half of the lake while there was a general increase in the eastern half of the lake [46]. These trends may lend support for the influences of changing thermal niche characteristics within Lake Superior, however there are many factors influencing population dynamics including stocking, food-web dynamics, and commercial and recreational fisheries harvest. Currently these organizations are implementing standardized methods and a synthesis of data across the 9 government and first nations research groups is currently underway (www.globalgreatlakes.org). At this time the data is not available to analyze lake wide range shifts for these species. The data for this type of analysis should be available in the near future and would yield valuable insights as to the realized effects of climate change on the spatial distributions of fishes in Lake Superior. This should be a research priority when a cross-agency synthesis exists.

In conclusion, our analysis finds significant changes in spatial extent and duration of preferred thermal habitat for four ecologically and economically important fishes in Lake Superior. Many modeling and projection studies have evaluated likely patterns in animal range shift and expansion [1], [23], [24]. The results presented here should be applicable to large lakes around the globe where warming may increase temperatures to approach thermal optimums for fish species. Monitoring should strive to evaluate fish populations in regions of rapid change. These changes in thermal habitat may intensify top down food web effects. Future studies should evaluate if predators are utilizing this increase in habitat, how this affects their abundance and consumption, and how consequent effects may cascade down to the lower trophic levels.
